# When Labour Moves

**Published:** 1924-09

**Authors:** 


### WHEN LABOUR MOVES.

At the end of six months of office of the first Labour Government in this country, we have been concerned to refresh our memory on the subject of the views of the Labour Party with regard to the health of the people. Those views are embodied in a statement of policy issued from Eccleston Square less than two years ago. It would be a chastening experience for the Minister of Health to read this pamphlet as part of his holiday literature. It cannot be denied that Mr. Wheatley's office has been much in evidence during the present Parliament. There has been much ado about Housing, and Mr. Wheatley has unquestionably established for himself a Parliamentary reputation. But, apart from a puff of smoke which had a familiar air in the House of Lords at the end of the session, we have looked in vain for some sign of the legislation in regard to the preventive and curative medical services which, in the opinion of the Labour Party, are so seriously overdue.

In the pamphlet to which we have referred the considered view of that party is set down in a statement that the entire reorganisation of the whole mechanism of both public and private medical services is urgently necessary. It is the aim of the party to have a complete public medical service which is to put every discovery of modern medical science, every class of specialist, every comfort of the best type of nursing home, sanatorium or hospital, within the reach of all, rich and poor, and, not least, of all the men and women who are neither the one nor the other. It is true that in this document the matter of the financing of so vast a scheme is dealt with in a somewhat casual way. The cost, we learn, would be a charge upon communal funds; this, however, would only be at the outset, and in due course the improved health of the workers would mean an increased production of wealth more than counter-balancing the whole cost of the health services. It is fair to say that the pamphlet expressly recognises that it might be impossible to carry the whole of such a programme into effect at once. Immediate progress in certain directions and along certain lines which would command fairly general agreement was, however, indicated as practical politics.

After making due allowance for the disillusioning chills of office, it is a little disappointing to find no evidence, in this pre-eminently important department of Health, of delivery or impending delivery of goods so freely and lavishly promised. We are aware that, where large and important interests are concerned, there must be consultation and discussion before Bills see the light. Even that would represent the one step with which the Prime Minister has told us that we must be satisfied. Of such preliminary conferences on Health matters there has reached us not so much as a whisper. The reference in the title of the pamphlet to a Labour movement seems to be something of a misnomer.

